# Relationships between Training Load, Salivary Cortisol Responses and Performance during Season Training in Middle and Long Distance Runners

**DOI:** 10.1371/journal.pone.0106066

**Published:** 2014-08-25

**Authors:** Carlos Balsalobre-Fernández, Carlos Mª Tejero-González, Juan del Campo-Vecino

**Affiliations:** Department of Physical Education, Sport and Human Movement, Autonomous University of Madrid, Madrid, Spain; University of the Balearic Islands, Spain

## Abstract

**Purpose:**

Monitoring training from a multifactorial point of view is of great importance in elite endurance athletes. This study aims to analyze the relationships between indicators of training load, hormonal status and neuromuscular performance, and to compare these values with competition performance, in elite middle and long-distance runners.

**Method:**

Fifteen elite middle and long-distance runners (12 men, 3 women; age = 26.3±5.1 yrs) were measured for training volume, training zone and session rate of perceived exertion (RPE) (daily), countermovement jump (CMJ) and salivary free cortisol (weekly) for 39 weeks (i.e., the whole season). Competition performance was also observed throughout the study, registering the season best and worst competitions.

**Results:**

Season average salivary free cortisol concentrations correlate significantly with CMJ (r = −0.777) and RPE (r = 0.551). Also, weekly averages of CMJ significantly correlates with RPE (r = −0.426), distance run (r = −0.593, p<0.001) and training zone (r = 0.437, p<0.05). Finally, it was found that the CMJ (+8.5%, g = 0.65) and the RPE (−17.6%, g = 0.94) measured the week before the best competition performance of the season were significantly different compared with the measurement conducted the week before the season’s worst competition performance.

**Conclusions:**

Monitoring weekly measurements of CMJ and RPE could be recommended to control training process of such athletes in a non-invasive, field-based, systematic way.

## Introduction

Although training volume is not directly related to performance of elite endurance athletes [1]–[3], it seems clear that such athletes need to train several hours per week during their training cycle to increase their performance [4]–[6]. Specifically, elite distance runners may run a lot of km throughout the season, with weekly amounts totaling up to 230 km or more in the case of marathon runners [7]. Thus, monitoring the training process of such athletes is essential in order to observe their adaptation to training load and to avoid overtraining syndrome [8]–[10]. Although the assessment of physiological parameters such as maximal oxygen uptake or blood cell count is of great importance in endurance sports [11], [12], their invasive, laboratory-based nature complicate regular measurement during daily training. Therefore, the use of some indicators that could, systematically and without disturbing the athletes, facilitate in-the-field monitoring of the training processes required.

The most common variable used on a daily basis to monitor the training process in running is the training load [9]. Specifically, training volume, intensity and session-RPE are the most used indicators of the training load because they can be assessed every day without disturbing the athletes and have shown significant relationships with performance or fatigue [13], [14]. For example, Esteve-Lanao et al. [13] recorded training volume and intensity of sub-elite cross-country runners for 6 months, discovering that the time expended training at low intensities (below the ventilator threshold) was significantly related to performance in a cross-country competition. Similarly, Garcin et al. [14] measured session-RPE in 8 young, elite middle-distance runners for 8 weeks, proving that this indicator of training load was able to detect states of overreaching. Meanwhile, the measurement of the vertical jump score as an indicator of neuromuscular performance has been used to assess fatigue in different kinds of athletes [15]–[17]. For example, it has been shown that the decrease in the countermovement jump (CMJ) score after a set of full-squats performed until failure correlates highly with blood lactate concentrations (r = 0.97, p<0.001) [17]. With respect to distance runners, it has been observed that a marathon competition significantly impairs the height reached in the CMJ [18]. Finally, the measurement of salivary free cortisol (hormone related with fatigue and stress) is commonly used to monitor the effects of training in several sports, due to its non-invasive, field-based nature [19], [20]. Moreover, it seems that cortisol levels are related to neuromuscular performance in well-trained strength athletes [21], [22]. For example, it has been demonstrated that changes in salivary free cortisol levels after 15 weeks of training are related to changes in the power clean mean power production over the same period in young elite wrestlers [23]. However, as far as we know, the relationship between salivary free cortisol levels and neuromuscular performance has not been studied on high-level middle and long-distance runners throughout a whole season.

Thus, although the measurement of the training load, salivary free cortisol or CMJ are very common to monitor training process in different kinds of athletes [7], [24], [25], any relationships between such variables in high-level distance runners, as well as their impact on the performance of such athletes, is, as far as we know, unknown. Therefore, the objectives of this investigation are: (1) to disclose the relationships between training load (measured using daily km run totals, training zone and session-RPE), salivary free cortisol and CMJ scores throughout a whole season in elite middle and long-distance runners; and (2) to compare the values of the study variables measured just before the season-best competition performance with the values measured just before the season-worst competition performance. As such, and according to the above, our study hypotheses are that: (a) weekly values of training load, salivary free cortisol and CMJ measured throughout the season are significantly related; and (b) the values of these variables measured just before the season-best competition performance are significantly different compared with those measured just before the season-worst competition performance.

## Materials and Methods

### Subjects

The participants in this study were 15 high-level middle and long-distance runners from the High Performance Sports Center Madrid (12 men, 3 women; age = 26.3±5.1 yrs), with personal bests in outdoor 1500-metres between 3∶38–3∶58 min. (men) and 4∶12–4∶23 min. (women). See Table 1 for more details.

**Table 1:** Characteristics of the participants (average ± SD).

	Age (yrs.)	Height (cm)	Weight (kg)	PB in outdoor1500 m (min:s)	PB in urban10 km (min:s)	Average CMJ(cm)	AverageSession-RPE(0–10)	Averageweekly kmrun	Averageweeklytraining zone	Average salivaryfree cortisol (ng/mL)
**Men**	25.7±5.4^N^	1.79±0.04^N^	63.9±3.1^N^	3∶48±0∶6^N^	30∶33±0∶43^N^	30.3±4.8^N^	5.7±0.4^N^	85.4±5.8^N^	1.8±0.06^N^	11.9±2.3^N^
**Women**	29.0±2.0^N^	1.67±0.05^N^	52.0±3.6^N^	4∶18±0∶5^N^	34∶50±1∶32^N^	27.9±1.4^N^	6.1±0.3^N^	83.2±7.0^N^	1.8±0.5^N^	12.4±2.1^N^

N = Normally distributed variable (Kolmogorov-Smirnov test, p>0.05). Abbreviations: PB = personal best; CMJ = countermovement jump; Session-RPE = session rate of perceived exertion.

Note: Training zones ranges from 1–3 according to session average running pace. Zone 1 = 3∶45–3∶10 min/km; Zone 2 = 3∶10–2∶50 min/km; Zone 3 = 2∶50 min/km to full sprint.

### Ethics statement

The study protocol complied with the Declaration of Helsinki for Human Experimentation and was approved by the ethics committee at the Autonomous University of Madrid (approval number CEI-45 889). Written informed consent was obtained from each subject before participation.

### Design

**Figure 1:**
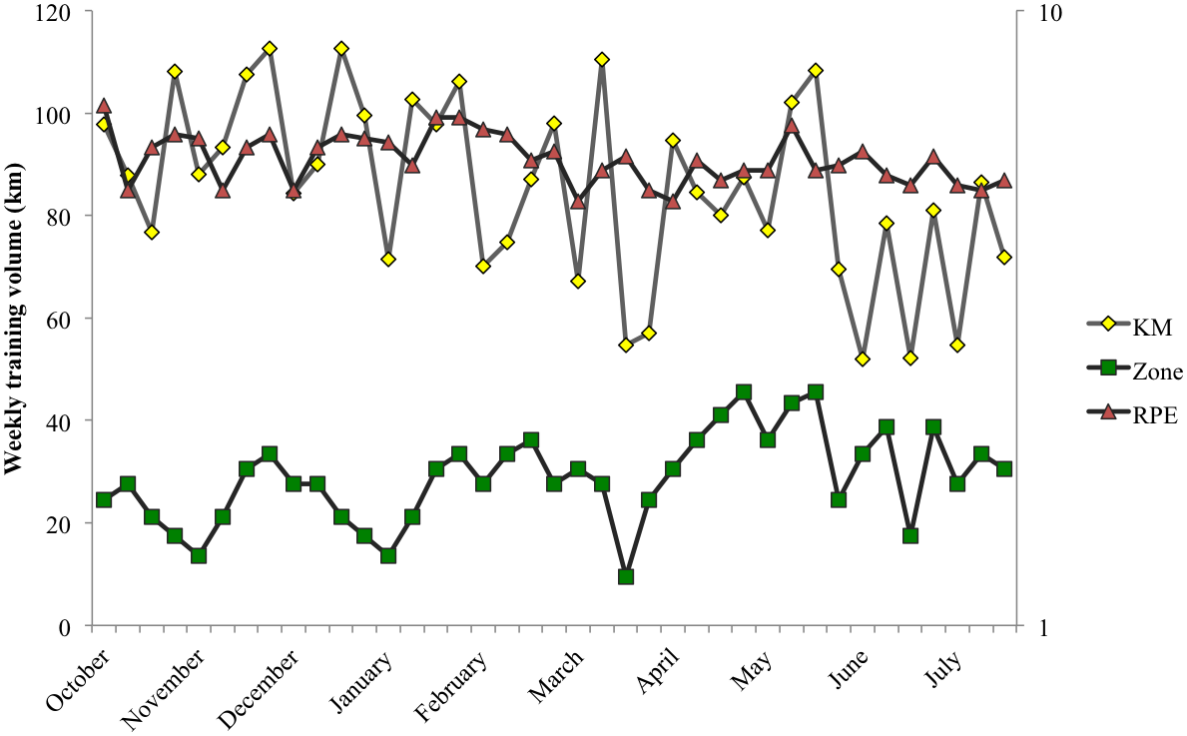
Training load variation throughout the whole season. Weekly training volume, training zone and session-RPE are represented. Training volume is represented on the left Y-axis (in km.), while training zone and session-RPE are represented on the right Y-axis with a logarithmic scale. Abbreviations: KM = weekly average km run; Zone = weekly average training zone; RPE = weekly average session-RPE.

Athletes were assessed for CMJ score, salivary free cortisol levels and training load throughout a whole season (October–July, 39 weeks). CMJ and cortisol were measured once a week, while training load, assessed by session rate of perceived exertion (session-RPE), km run and training zone were measured daily. Competition performance was observed throughout the whole season, registering the season best (SB) and worst (SW) results (i.e., fastest and slowest times in competitions). Correlations between the variables evaluated in this investigation and differences in CMJ, cortisol and training load just before the SB and SW events were then analyzed. See Figure 1 for more information about the training load variation throughout the season.

### Methodology

#### Training load

Training load was measured daily throughout the whole season using daily session-RPE, km run and training zone parameters. Session-RPE was assessed after each training session using the Borg 0–10 scale by asking, “How hard was the training session, with 0 being very, very light and 10 extremely heavy?”. Kilometres run and training zone were recorded using the coach’s endurance training programmes designed for each athlete. When an athlete coudln’t fulfil his or her training programme, the km run and training zone values of the what the athlete did complete were recorded. Every training session was classified in one of 3 training zones according to the characteristics of the principle part of the session: Zone 1 included long-distance continuous training, or interval training with long sets (4–6 km), at paces of between 3∶45-3∶10 min/km; Zone 2 included middle-distance interval training (sets of 1–3 km) at paces between 3∶10-2∶50 min/km; and Zone 3 included short-distance and sprint interval training (sets of 200–600 m) at paces ranging from 2∶50 min/km to full sprint.

#### Salivary-free cortisol

To establish the basal cortisol level (in ng/mL), athletes collected a saliva sample immediately after they woke up (i.e., 1–2 min after waking up), with an empty stomach, once a week throughout the whole season using Salivette tubes (Sarstedt, Germany). Athletes chewed the cotton inside the Salivette tube for 60 seconds after they rinsed their mouth with water, then they stored the sample at −20°C for 1 hour before subsequently bringing it to the laboratory for analysis. All measurements were performed on the same day of the week, at the same time and under the same environmental conditions. All the subjects woke up almost at the same time of the day (8∶30–9 a.m.) since they lived in the same area and trained together. The samples were then stored at −20°C according to the manufacturer’s instructions. All samples were analyzed at the Biochemical Laboratory of the Polytechnic University of Madrid (Official Lab. Number 242 in the Region of Madrid) using Free Cortisol in Saliva ELISA Assay kits (Demeditec Diagnostics, Germany). The coefficient of variation (CV) of the measurements was CV = 4.3–5.6%.

#### CMJ

The CMJ scores were measured once a week throughout the whole season, on the same day that saliva samples were collected and just before beginning the training session. An Optojump infrared (IR) platform (Microgate, Italy) was used for the assessment. The CMJ was performed with hands on hips, knees straight in the flight phase while trying to jump as high as possible. All measurements were taken on the same day of the week, at the same time and under the same spatial and environmental conditions. The average of 3 attempts was recorded. The reliability of the measurements was calculated using the intraclass correlation coefficient (ICC = 0.979–0.990, p<0.001).

### Statistical analysis

To analyze the relationship between the variables, we used the Pearson correlation coefficient, unilateral contrast. For the comparison of means, we used the paired samples t-test. For the calculation of the effect size (ES), we used the Hedge’s g. Effects sizes below 0.5 were considerate *low,* and ES between 0.5–1.0 were considerate *moderate to high* [26]. The level of significance was set at 0.05. All calculations were performed using IBM SPSS Statistics 22 software (IBM Co., USA).

## Results

Significant correlations were found between season average CMJ and cortisol (r = −0.777, p<0.001), CMJ and session-RPE (r = −0.489, p = 0.049) and session-RPE and cortisol (r = 0.551, p = 0.025) values. Analysis of the average weekly values of the variables throughout the whole season showed that CMJ scores correlate significantly with session-RPE (r = −0.426, p = 0.012), cortisol (r = 0.556, p<0.001), km run (r = −0.593, p<0.001) and training zone (r = 0.437, p = 0.007). Also, km run correlates significantly with session-RPE (r = 0.444, p = 0.009) and cortisol levels (r = −0.366, p = 0.017). See Table 2 for more details.

**Table 2:** Pearson correlation coefficient (r) between study variables.

	Session-RPE		Cortisol		Km run		Training zone	
	Season average	Weekly average	Season average	Weekly average	Season average	Weekly average	Season average	Weekly average
**CMJ**	−**0.489** *	−**0.426** *	−**0.777** **	**0.556** **	0.133	−**0.593** **	−0.231	**0.437** *
**Session-RPE**	–	–	**0.551** *	−0.037	0.168	**0.444** *	−0.130	−0.080
**Cortisol**			–	–	−0.051	−0.366	−0.228	0.171
**Km run**					–	–	−**0.599** *	−0.044

Season average: correlations between average variables for the group throughout the whole season; Weekly average: correlations between average variables of the group for every week of the season. CMJ = countermovement jump score; Session-RPE = rate of perceived exertion of the training session; km run = total weekly km run.

*p<0.05;

**p<0.001.

Comparing the values for the variables measured the week before the season-best (SB) and season-worst (SW) competition performances, it was found that the CMJ scores prior to the SB was significantly higher than the CMJ score prior to the SW (+8.5%, g = 0.65, p<0.001). The session-RPE for the week before the SB was significantly lower than the session-RPE for the week before the SW (−17.6%, g = 0.94, p = 0.022). There were no significant differences between salivary free cortisol, km run or training zone values measured before SB and SW. See Table 3 for more details.

**Table 3:** Comparison of variables measured the week before the season best (SB) and season worst (SW) competition performances.

Variables	SAv	SB	SW	Hedge’s g	95% CI	%
	Av ± SD	Av ± SD	Av ± SD			
CMJ (cm)	29.8±4.6^N^	32.5±4.5^N^	29.7±4.0^N^	0.65	[1.9, 3.6]	+8.5**
Session-RPE (0–10)	5.8±4.5^N^	5.6±1.3^N^	6.6±0.8^N^	0.94	[−1.9, −0.2]	−17.6*
Salivary free cortisol (ng/mL)	12.0±2.2^N^	15.7±7.3^N^	12.1±6.7^N^	0.52	[−1.2, 8.3]	+22.9
Km run	84.9±5.9^N^	75.8±24.4^N^	87.2±22.8^N^	0.48	[−26.8, 4.2]	−15.0
Training zone	1.8±0.8^N^	1.9±0.4^N^	2.0±0.4^N^	0.21	[−0.4, 0.2]	−5.2

N = Normally distributed variable (Kolmogorov-Smirnov test, p>0.05);

*p<0.05;

**p<0.001.

Abbreviations: SAv = average value for the season; SB = value measured the week before the season best competition performance; SW = value measured the week before the season worst competition performance; Av = average value; SD = standard deviation; 95% CI = 95% confidence interval of the differences between SB and SW values; % = percentage difference between SB and SW; CMJ = countermovement jump score; Session-RPE = rate of perceived exertion of the training session; km run = total weekly km run.

## Discussion

The results of our study have revealed that noteworthy relationships exist between salivary free cortisol and CMJ scores assessed throughout a whole season in elite middle and long-distance runners. Also, moderate relationships between session-RPE and salivary-free cortisol were found. Firstly, our data demonstrates a significant trend in which athletes with higher [average] cortisol levels measured throughout the season were those with lower CMJ scores. It worth to mention that females and males salivary free cortisol were equivalent throughout the season, despite some studies have reported hormonal differences between sexes [27], [28]. It has previously been demonstrated that post-exercise blood lactate and ammonia concentrations are negatively and significantly related to the height jumped in the CMJ performed just after an intensive exercise session, so greater CMJ decreases correspond to a higher level in such physiological markers [17]. In highly-trained strength athletes, it was observed that salivary cortisol is negatively and significantly related to neuromuscular performance [14], [22]. For example, Kraemer et al. [22] studied the changes on cortisol and performance of a group of highly-trained soccer players throughout a season, and they showed that salivary cortisol levels measured before training have a significant correlation with the vertical jump height scores recorded on the same day (r = −0.59, p<0.05). That study concludes that athletes starting the season with elevated cortisol values may experience significant reductions on performance during the season. Our study expands the knowledge in this respect, demonstrating that subjects with higher long-term salivary free cortisol levels significantly tend to be those with lower CMJ scores throughout the season. However, when correlations between weekly average cortisol and CMJ values were analyzed, a significant trend was observed in which the weeks with higher salivary free concentrations were those in which higher CMJ scores were recorded. Thus, despite athletes with higher [average] cortisol levels throughout the season had significantly lower CMJ [average] values, it seems that weeks with higher cortisol concentrations produce a potentiation of CMJ performance. Although some investigations have studied the relationships between salivary cortisol concentrations and force production [23] or vertical jump [21], they used strength-related athletes and measured the variables less frequently (6 times during a season). However, we are not aware of studies which analyze the relationship between weekly salivary free cortisol concentrations and CMJ height measurements throughout a whole season in elite middle and long-distance runners. Further research is needed to clarify the nature of the relationship between salivary cortisol concentrations and CMJ performance in elite middle and long-distance runners.

Furthermore, training load was also shown to correlate significantly with salivary cortisol levels and CMJ scores. Specifically, weekly values for session-RPE, km run and training zone correlate significantly with CMJ scores, in such a way that in weeks with lower rates of perceived exertion, less km run and with higher training zone (i.e., more Zone 3 sessions) correspond with those weeks with higher CMJ performance. Similarly, our data shows that athletes with higher average season-long, session-RPE values significantly tend to be those who trained more km and had higher average season-long salivary cortisol concentrations. Some authors have proposed that elite athletes endurance training must consist of lower training volume to produce the desired adaptations [1], [29]. For example, it has been proven in elite kayakers that a 1-year traditional endurance training programme produces lower increases in physical fitness than a 1-year block-periodisation endurance training programme with 50% less volume [1]. Similarly, it has been shown that resistance training performed until failure produces higher fatigue accumulation and lower increases in performance than an identical training regime in which half of the possible repetitions per set were executed [29]. Therefore, results in our study show that training with a higher volume and greater session-RPE correlate significantly with higher salivary cortisol concentrations and a lower performance in the CMJ.

Moreover, when analyzing the difference between the training load, salivary free cortisol and CMJ values measured the week before the season best and season worst competition performances, it is observed that, before the SB, athletes achieved higher CMJ scores than before the SW. Also, the CMJ before the SB was significantly higher than the season average, while the CMJ measured before the SW did not vary from the season average. Meanwhile, session-RPE measured before the SB was significantly lower than that measured before the SW. Furthermore, session-RPE before the SB was lower (although not significantly) than the season average, while the session-RPE before the SW was significantly higher than the season average. There were no significant differences in the other variables, although athletes trained 11.4 km less the week before the SB than before the SW performances. That is to say, the week before the best competition performance of the season, athletes trained with significantly lower session-RPE, achieved higher CMJ scores and ran more than 11 km less in comparison with the SW performance. In this sense, it was previously demonstrated that the reduction of training volume near to an important competition could improve physical performance of highly-trained athletes [30], [31].

In summary, the weekly assessment of training load (using daily session-RPE, km run and training zone), salivary free cortisol and CMJ scores may help to control the training process of elite middle and long-distance runners using simple, non-invasive, systematic, field-based methods throughout a whole season. For the very first time, this study analyzes the relationships between training load, salivary free cortisol concentrations and CMJ scores measured throughout 39 weeks of training by such athletes.

## Practical Applications and Conclusions

This study reveals the significant relationships between average season values for CMJ scores, salivary free cortisol levels and session-RPE-athletes with higher salivary cortisol concentrations demonstrated a significant correlation with a tendency for lower CMJ scores and higher session-RPE values-. When observing the weekly average of the group, it was observed that the weeks in which higher CMJ scores were achieved significantly correspond to those with lower volume (km), higher training zone and lower session-RPE values. Finally, it was proven that CMJ scores were significantly higher and session-RPE values were significantly lower the week before the season best competition performance in comparison with the week before the season worst competition performance. The results of our study agree with those in other investigations which propose that training with lower volumes and less fatigue-inducing sessions are more effective in terms of increasing performance. Monitoring training load through daily session-RPE and weekly CMJ measurements could help control the training process of elite middle and long-distance runners. Furthermore, such variables can be measured throughout a whole season without interfering with the athlete’s training using simple, non-invasive, field-based methods.
